# Silver nanoparticles alter the dimerization of Aβ_42_ studied by REMD simulations†

**DOI:** 10.1039/d4ra02197e

**Published:** 2024-05-08

**Authors:** Quynh Mai Thai, Phuong-Thao Tran, Huong T. T. Phung, Minh Quan Pham, Son Tung Ngo

**Affiliations:** a Laboratory of Biophysics, Institute of Advanced Study in Technology, Ton Duc Thang University Ho Chi Minh City Vietnam ngosontung@tdtu.edu.vn; b Faculty of Pharmacy, Ton Duc Thang University Ho Chi Minh City Vietnam; c Hanoi University of Pharmacy 13-15 Le Thanh Tong Hanoi Vietnam; d NTT Hi-Tech Institute, Nguyen Tat Thanh University Ho Chi Minh City Vietnam; e Institute of Natural Products Chemistry, Vietnam Academy of Science and Technology Hanoi Vietnam; f Graduate University of Science and Technology, Vietnam Academy of Science and Technology Hanoi Vietnam

## Abstract

The aggregation of amyloid beta (Aβ) peptides is associated with the development of Alzheimer's disease (AD). However, there has been a growing belief that the oligomerization of Aβ species in different environments has a neurotoxic effect on the patient's brain, causing damage. It is necessary to comprehend the compositions of Aβ oligomers in order to develop medications that may effectively inhibit these neurotoxic forms that affect the nervous system of AD patients. Thus, dissociation or inhibition of Aβ aggregation may be able to prevent AD. To date, the search for traditional agents and biomolecules has largely been unsuccessful. In this context, nanoparticles have emerged as potential candidates to directly inhibit the formation of Aβ oligomers. The oligomerization of the dimeric Aβ peptides with or without the influence of a silver nanoparticle was thus investigated using temperature replica-exchange molecular dynamics (REMD) simulations. The physical insights into the dimeric Aβ oligomerization were clarified by analyzing intermolecular contact maps, the free energy landscape of the dimeric oligomer, secondary structure terms, *etc.* The difference in obtained metrics between Aβ with or without a silver nanoparticle provides a picture of the influence of silver nanoparticles on the oligomerization process. The underlying mechanisms that are involved in altering Aβ oligomerization will be discussed. The obtained results may play an important role in searching for Aβ inhibitor pathways.

## Abstract 2

The dimerization of the Aβ_42_ peptide is inhibited by the silver nanoparticle.

## Graphical abstract

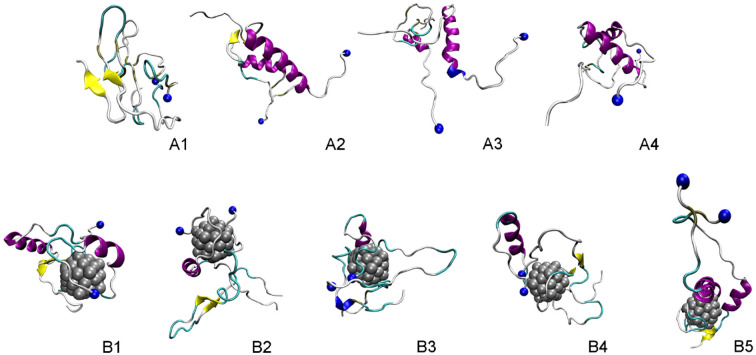

## Introduction

The self-assembly of Aβ peptides into oligomers^1,2^ is associated with AD,^3^ which is one of the most common dementias.^4,5^ In 2023, more than 11 million Americans provided 18.4 billion working hours in order to take care of about 6.9 million elders who are living with AD.^6^ $346.6 billion is the cost of health, long-term care, and hospice services for these elders. Despite several previous research studies,^7–12^ the mechanism of AD pathogenesis is unidentified, resulting in the failure of treatment.^12–14^ The pathogenic mechanism of AD has not been determined despite intensive and extensive studies,^15^ which hinders the prevention and treatment of the disease. The patient's brain is slowly destroyed, which reduces cognition and life skills.^16^ Many works have indicated a wide variety of probable reasons for AD, which can be grouped into three categories, including molecular, cellular, and genetic imbalances. For example, Ca^2+^ homeostasis falls under the category of cellular imbalances, while genetic imbalance is associated with DNA damage. Here we focus on molecular imbalances, including tau,^17^ amyloid,^18^ and cholinergic^19,20^ hypotheses. Among these, numerous pieces of evidence indicate that the oligomerization of Aβ peptides into extracellular transient oligomers plays a role as neurotoxicity agents causing AD.^18,21,22^ The main types of Aβ peptides are Aβ_40_ and Aβ_42_ with 40 and 42 residues,^23^ respectively. It should be noted that the structural Aβ peptides are very flexible in solution, resulting in a lack of stable conformations. Aβ peptide is a molecule having low hydrophobicity, a high net charge, and a few aggregation-prone regions.^24^ The Aβ oligomerization is highly sensitive to the sequences^25,26^ and is associated with AD hallmarks, including the amyloid hypothesis.^27–30^

The amyloid hypothesis states that the aggregation of Aβ is causally linked to the development of AD. Recently, there has been a growing belief that the oligomerization of Aβ species in different environments has a neurotoxic effect on the patient's brain, causing damage.^31,32^ Characterizing the shapes of Aβ oligomers is thus required to develop agents to prevent the neurotoxic forms.^33–36^ Several investigations are thus targeted on clarifying the formation of Aβ in solution *via* both MD and REMD simulations.^37–39^ In particular, the structural properties of the Aβ_16–35_ fragment were studied as a model for the Aβ peptide *via* REMD simulations.^40^ The influence of mutations on the folding process of dimeric and monomeric forms of Aβ peptides was clarified.^41^ The probability of the solvated Aβ_40/42_ peptide forming tetrameric β-barrel structures was determined.^42^ Therefore, computational approaches were extensively and intensively used for studying the influence of various inhibitors on the conformations of Aβ peptides.^43^ In particular, the stronger inhibitors normally form larger binding affinity Aβ peptides. The good inhibitors also prevent the formation of β-structure effectively. Numerous compounds were suggested to be able to inhibit the Aβ oligomerization, such as β-sheet breaker peptides,^37^ curcumin,^44^ epigallocatechin gallate,^45^ astaxanthin,^34^ resveratrol,^46^*etc.* Unfortunately, there is no cure for AD based on small compounds targeting Aβ peptides.^14^ However, recent AD therapeutic developments are associated with antibody drugs against the aggregates.^47,48^

**Fig. 1:**
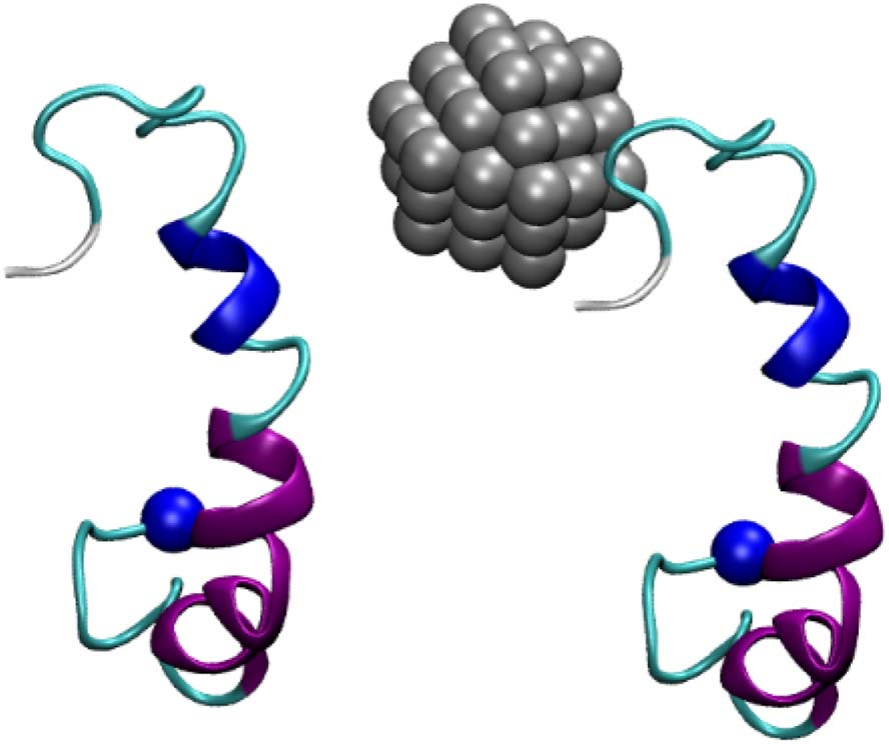
Initial conformation of Aβ_42_ dimer + Ag_55_ nanoparticle. The distance between the Aβ peptides and nanoparticle is larger than 1.2 nm. The blue balls mention the N-terminal of the Aβ peptide. The solvation molecules were hidden for clarifying.

Nanoparticles are particles with physical, chemical, or biological effects whose sizes are within the nanoscale range (1–100 nm).^49^ Nanoparticles can be used for a variety of pharmaceutical and medical applications, such as drug delivery, imaging, and cancer therapy.^50^ In this context, nanoparticles emerge as highly potent substrates that may be used to treat AD by preventing the oligomerization of Aβ peptides (*cf.*Fig. 1). The 11-mercapto-1-undecanesulfonate-coated gold nanoparticles have been informed to be able to spot the generation of amyloid fibrils derived from various amyloidogenic proteins.^51^ These nanoparticles are powerful tools for investigating amyloid morphologies, using cryogenic transmission electron microscopy (cryo-EM).^51^ Moreover, silver nanoparticles are also small particles of silver that have been reduced to a size of less than 100 nanometers. They have a number of unique properties, including the ability to kill bacteria and viruses. This makes them useful for a variety of applications, including wound dressings, food preservation, and water purification.^52^ Similar to gold nanoparticles, silver nanoparticles can induce fast disintegration of the fibrils. Triangular silver nanoplates stabilized with poly(vinyl)pyrrolidone and carrying a negative charge were more efficient compared to silver nanospheres stabilized with poly(vinyl)pyrrolidone.^53,54^ In particular, it was demonstrated that when Aβ fibrils were exposed to triangular silver nanoplates and subjected to near infrared illumination, the fibrils were dissolved in just 1 hour. In contrast, it took approximately 70 hours for the nanospheres to achieve the same result. Silver nanoparticles were used to monitor the dynamical behavior of Aβ_25–35_ peptides.^55^ The silver nanoparticles were thus suggested that they are able to a good therapy solution to prevent amyloidosis diseases such as AD.^55^

In this context, studying the influence of nanoparticles on the oligomerization of Aβ peptides is of great interest. Therefore, in the project, we propose to use atomistic simulations to assess the folding process of Aβ peptides wild-type under the effects of silver nanoparticle. In particular, the structural change, dynamic behavior, and kinetics of the Aβ oligomerization in the presence and absence of nanoparticles will be clarified.

## Materials and methods

### The Aβ_42_ dimer and Aβ_42_ dimer + silver nanoparticle in solution

The initial conformation of the monomeric Aβ_42_ peptide can be downloaded from the Protein Data Bank (PDB) with PDB ID 1Z0Q.^56^ The aqueous solution structure of Aβ_42_ was often used to probe the interaction between Aβ and different molecules in solution.^57^ The Aβ peptides are randomly inserted into a dodecahedron box with the minimum distance between various monomers larger than 12.0 Å. The silver nanoparticle with 55 atoms is generated *via* the nanomaterial modeler tools *via* CHARMM GUI^58^ according to the stable structures of nanoparticles, referring to the previous work.^59^ In particular, the interface force field (IFF)^60^ was employed to present the silver nanoparticle. It should be noted that IFF facilitates using computational methods to probe biomaterials and advanced materials. Using IFF, the interaction between nanomaterials and proteins was successfully characterized, including silver nanomaterials and SARS-CoV-2 receptor-binding protein.^61^ The Aβ peptides are parameterized *via* CHARMM36m force fields.^62^ The TIP3P water model is employed to describe the water molecule.^63^ The interface force field^60^ will then be used to parameterize the silver nanoparticle. An example of Aβ_42_ dimer and Aβ_42_ + silver nanoparticle is shown in Fig. 1. In particular, both complexes were inserted into the dodecahedron box with a volume of 584 nm^3^, respectively, and the systems consist of *ca.* 56 300 atoms totally.

### MD simulations

The solvated Aβ peptides in the presence and absence of nanoparticles were simulated using GROMACS version 2019.6.^64^ In particular, the MD simulations utilize the leap-frog stochastic dynamics integrator with a time step of 2 fs. The pressure of simulation was selected as 1 bar. A relaxation time of 0.1 picoseconds is selected. The V-rescale thermostat and Parrinello–Rahman barostat were used for temperature and pressure simulations.^65,66^ The LINCS^67^ approach restricts all covalent bonds to a fourth order. The non-bonded interaction pair list is updated using a 0.9 nm cut-off every 10 femtoseconds. The rapid and efficient particle-mesh Ewald electrostatics method is used to calculate the interactions between charged particles, with a cutoff distance equal to the range of non-bonded interactions. The van der Waals (vdW) interactions are calculated using a cut-off distance of 0.9 nm. In the first step, energy minimization using the steepest descent protocol was performed. The minimized systems were formerly relaxed in NVT and NPT ensembles with the positionally restrained condition using a weak harmonic potential (100 ps each). The last snapshots of NPT simulations were then used as the initial structures for REMD simulations at different temperatures.

### REMD simulations

Temperature REMD simulation^68^ has been widely used to investigate the structural change of disordered proteins, including Aβ systems.^69–71^ The last snapshots of NPT simulations (mentioned above) were used as initial conformations of REMD simulations. The temperature REMD simulations with a length of 500 ns will be carried out as previously described^72^ using 44 temperatures ranging from 308.50 to 384.33 K (*cf.* the ESI†). In particular, the temperatures were generated *via* a web-server generator.^73^ Atomic coordinates and other data (energy and velocity, *etc.*) will be recorded every 10 ps during REMD simulations to analyze the structural changes of the systems.

### Structural analysis

The free energy landscapes (FEL) of the Aβ systems over the equilibrium region of REMD simulations will be obtained using the GROMACS tool “gmx sham”,^74,75^ in which the radius of gyration (*R*_g_) and root mean square deviation (RMSD) will be used as the reaction coordinates. Moreover, the FEL will be possibility constructed *via* the principal component analysis (PCA) method, with the first and second principal components being reaction coordinates.^76^ In combination with the FEL results, the clustering method will be used to obtain the representative conformation of the considered systems.^77^ IMPACT tools were used to calculate the collision cross-section (CCS) of Aβ peptides.^78^ The intermolecular non-bonded contacts were counted when the minimum distance between non-hydrogen atoms of different Aβ residues to Aβ residues or to nanoparticles was smaller than 4.5 Å. The intermolecular hydrogen bond (HB) between the Aβ residues and the Aβ residues/nanoparticles was endorsed when the angle ∠ acceptor (A)–hydrogen (H)–donor (D) is larger than 3π/4 and the pair A–D is smaller than 3.5 Å. The secondary structure of Aβ peptides can be predicted *via* the Dictionary of Protein Secondary Structure (DSSP).^79^

## Results and discussion

The conformational change of Aβ peptides was popularly investigated *via* REMD simulations, which is one of the most effective enhanced sampling approaches.^80^ In particular, the large difference in temperature range can increase the efficiency of the approach compared with conventional MD simulations.^81,82^ The temperatures were selected in the range from 308.50 to 384.33 K by using a web-server generator.^73^ The exchange rates diffused in the range from 21 to 24% implying the efficiency of the REMD simulations. The superposition of computed values over the various intervals mentions the simulating convergence (Fig. S1 and S2 of the ESI file†). The REMD simulations were performed over 500 ns of MD simulations, which resulted in *ca.* 44 μs of MD simulations totally. The systemic coordinates at 310 K were recorded every 10.0 ps.

**Fig. 2:**
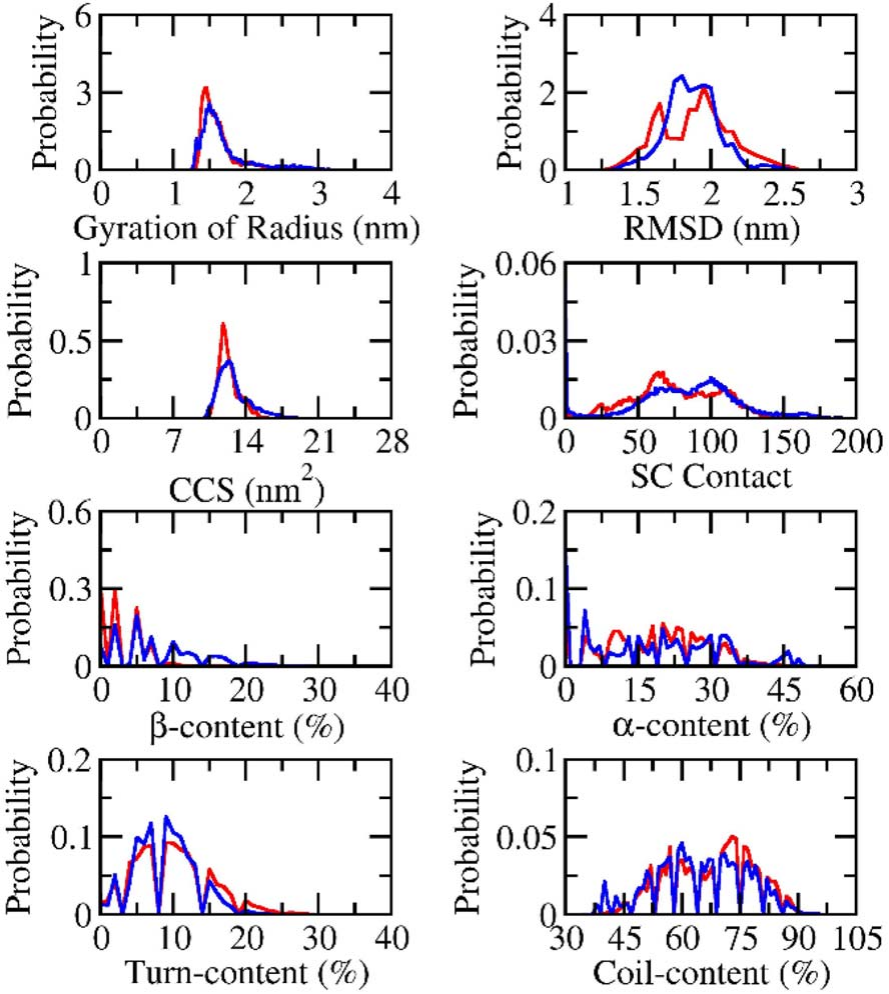
Computed metrics of dimeric Aβ_42_ in the presence (red curve) and absence (blue curve) of the silver nanoparticle.

The structural change of dimeric Aβ peptides in the presence and absence of silver nanoparticle was monitored and analyzed (*cf.*Fig. 2) over equilibrium domains, which ranged from 260 to 500 ns in the simulations. The radius of gyration, *R*_g_, of dimeric Aβ_42_ peptides without the presence of silver nanoparticle diffuses in the range from 1.26 to 3.56 nm with a mean value of 1.70 ± 0.37 nm. The value is slightly larger than that interacting with silver nanoparticle, which varies in the range from 1.30 to 3.14 nm with an average value of 1.60 ± 0.23 nm. The RMSD value of the isolated Aβ dimer ranges from 1.15 to 2.90 nm with a mean value of 1.87 ± 0.18 nm. While, the dimeric Aβ peptides in the presence of Ag_55_ forms RMSD in the range from 1.30 to 2.70 nm, with an average value of 1.89 ± 0.25 nm. The presence of silver nanoparticle alters the CCS curve of the dimeric Aβ_42_ (*cf.*Fig. 2). The mean CCS values of the dimers are changed, among these, the dimers with and without silver nanoparticle form an average CCS of 12.77 ± 1.50 and 12.32 ± 0.96 nm^2^, respectively. The total number of sidechain (SC) contacts between different residues of different chains is also changed. The silver nanoparticle significantly alters the SC contact between two monomers. The mean of SC contacts is 75.03 ± 30.41 and 81.76 ± 36.70 nm^2^ corresponding to the dimeric Aβ_42_ in the absence and presence of Ag_55_, respectively.

The secondary structure terms were also investigated *via* the Define Secondary Structure of Proteins (DSSP) protocol.^79,83^ The obtained results are presented in Fig. 2. In details, the β-content changes from 0 to 33% with an average of 8.2 ± 5.7% and from 0 to 26% with a mean value of 3.5 ± 3.8% in the absence and presence of Ag_55_. The free Aβ_42_ dimer in the present work adopts more and less β-content compared with the previous works by Barz *et al.*,^84^ which occupied 5.6 ± 0.7% and Maryam *et al.*,^85^ which formed 13%. The α-content of the free Aβ_42_ dimer varies in the range from 0 to 51% with a mean value of 18.2 ± 13.3%. The appearance of Ag_55_ turns the metric to 19.0 ± 10.3% and ranges from 0 to 48%. In similar, the Ag_55_ increases the turn-content of Aβ_42_ dimer from 8.9 ± 4.1 to 9.9 ± 5.2%. The coil-content of Aβ_42_ dimer increases from 64.7 ± 11.7 to 67.6 ± 10.9% *via* the influence of Ag_55_. Besides, secondary structure terms fall in a large range mentioning a broad structural change observed over the REMD simulations.

**Fig. 3:**
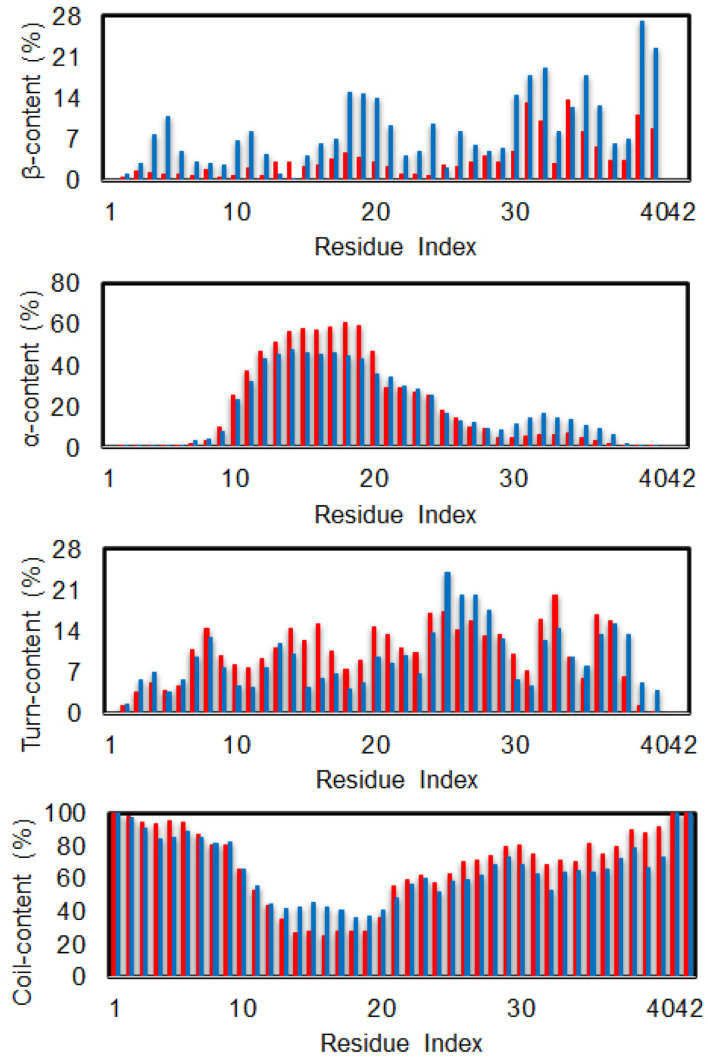
Secondary structure per residues of the dimeric Aβ_42_ in the presence (red bar) and absence (blue bar) of Ag_55_.

The secondary structure terms over individual residues were also reported (*cf.*Fig. 3). The outcomes are in good consistent with the previous investigation *via* MD simulation using the same force field.^85^ In good agreement with the total metrics above, almost dimeric residues adopt a less β-content when silver nanoparticle was induced. Moreover, the N-terminal of the dimer significantly increases α-content with the presence of Ag_55_, whose residues range from 10–20. Correspondingly, the coil-content of the sequence 10–20 was significantly decreased when Ag_55_ was induced. Turn-content population density per residues was also altered as shown in Fig. 3. The outcomes are similar results to the secondary structure of the Aβ_40_ monomer in binding mode with gold nanoparticle.^86^ In addition, the local secondary structure term of a residue along sequence of the dimeric Aβ was thus reported, which is in good consistent with the previous work.^87^

**Fig. 4:**
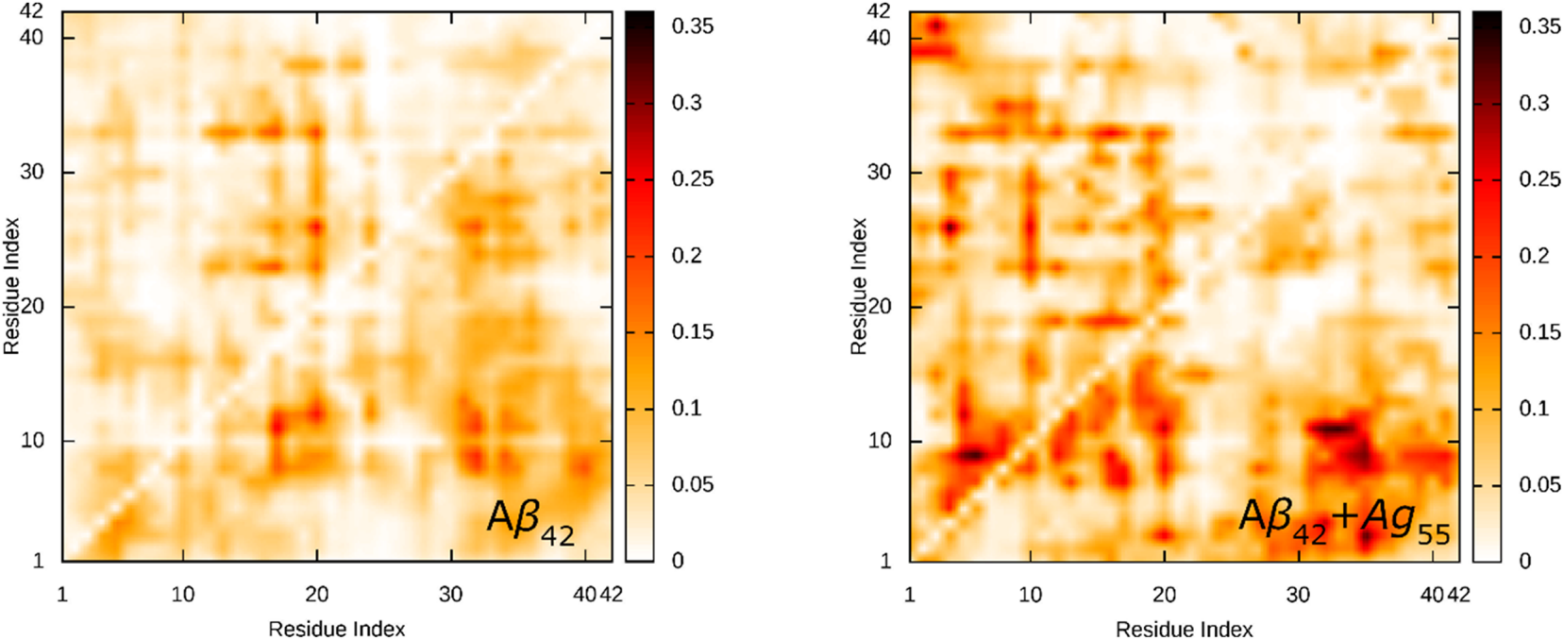
SC contact maps between individual chains of the dimeric Aβ_42_ and Aβ_42_ + Ag_55_.

The SC contact maps between individual chains of the dimeric Aβ_42_ and Aβ_42_ + Ag_55_ were generated and shown in Fig. 4. Interestingly, the appearance of Ag_55_ enhanced the binding between different residues of various chains of the dimeric Aβ_42_ peptide. The obtained results are in good consistent with the analysis of the total number of SC contact between different chains. It may be argued that the Ag_55_ nanoparticle force the dimer to stick together.

**Fig. 5:**
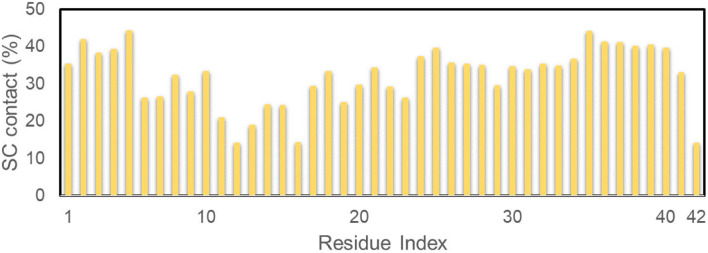
SC contact distribution between individual chains of the dimeric Aβ_42_ and Ag_55_.

The referential binding site of Ag_55_ to the dimeric Aβ_42_ can be probed by computing the distribution of SC contact between Ag_55_ and the dimeric Aβ_42_. In particular, the SC contact was counted when the spacing between non-hydrogen atoms of Aβ_42_-specific residues and Ag_55_ was smaller than 0.45 nm. The obtained results are shown in Fig. 5. Ag_55_ forms SC contacts to two domains, including sequences 2–5 and 35–40, over more than 38% of considered snapshots. It may be argued that the silver nanoparticle prefers to bind to these domains.

**Fig. 6:**
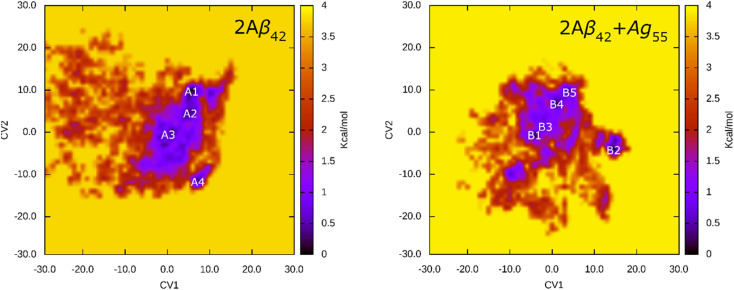
FEL of 2Aβ_42_ and 2Aβ_42_ + Ag_55_ systems over an interval of 260–500 ns of REMD simulations at 310 K. In particular, the first and second principal components were utilized as two reaction coordinates.

The FEL of dimeric Aβ_42_ with and without Ag_55_ was produced over the interval 260–500 ns of REMD simulations at 310 K. 24 000 snapshots of each dimeric Aβ_42_ system were used for PCA analysis. The representative structure of dimeric Aβ_42_ with and without the presence of Ag_55_ nanoparticle was then obtained *via* the clustering method.^77,88^ The FEL was obtained and shown in Fig. 6. Absolutely, the appearance of silver nanoparticle modifies the FEL of the dimeric Aβ_42_ peptide. The number of minima was also altered. Among these, the isolated Aβ_42_ dimer forms 4 minima denoted as A1, A2, A3, and A4, while the Aβ_42_ dimer + Ag_55_ adopts 5 minima denoted as B1, B2, B3, B4, and B5. Every dimeric Aβ_42_ structures located in the minima was collected to evaluate the representative conformation *via* the clustering approach on the backbone with a cutoff of 0.03 nm.

**Fig. 7:**
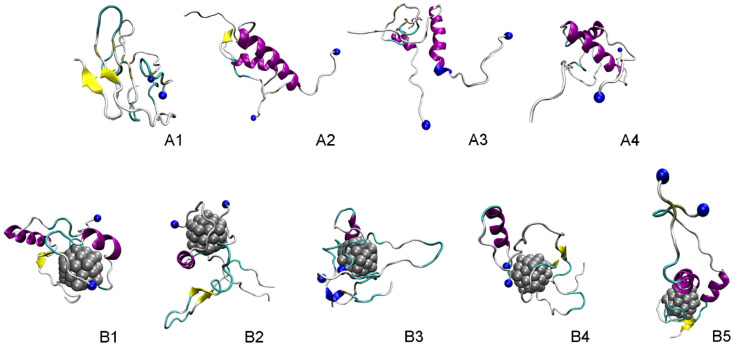
Representative structures of the dimeric Aβ_42_ and Aβ_42_ + Ag_55_, which located in the corresponding minima of FELs. In particular, yellow color indicates β-content. Purple and blue colors imply α-helix and 3–10 helix structures, respectively. Blue ball mentions the N-terminal of Aβ.

The representative structures of the dimeric Aβ_42_ and Aβ_42_ + Ag_55_ were obtained and shown in Fig. 7. Among these, A1–A4 correspond to 4 minima of the isolated dimeric Aβ_42_ system, which coordinates (CV1, CV2) at (7.0, 9.0), (5.0, 4.0), (0.5, −3.5), and (8.5, −12.5), respectively. The population of these minima is 12, 10, 12, and 4%, respectively. Besides, the conformations B1–B5 correspond to 5 minima of the dimeric Aβ_42_ + Ag_55_, which coordinates (CV1, CV2) at (−4.5, −1.5), (13.5, −4.5), (−3.5, 0.5), (0.5, 6.0), and (3.5, 9.5), respectively. The population of these conformation is of 8, 10, 7, 10, and 7%, respectively. In good agreement with the whole trajectory analysis above, the dimeric Aβ_42_ adopts more coil-structure when Ag_55_ was induced. It may be argued that the silver nanoparticle forms strong effects on the conformation of the dimeric Aβ_42_.

## Conclusions

In this work, the folding process of the dimeric Aβ_42_ with and without the presence of Aβ_42_ was investigated *via* extensive REMD simulations. Several conformations of the dimers were produced and recorded.

The structural metrics including RMSD, *R*_g_, CCS, and secondary structure terms vary in a large range implying the reality of the simulations. In particular, the appearance of silver nanoparticle rigidly alters the structure of the dimeric Aβ_42_. The β-content was significantly reduced when Ag_55_ was induced. Inversely, the α-, turn-, coil-content of the dimer was increased. Moreover, the size of the dimer is slightly increased due to the influence of silver nanoparticle. However, the number of SC contacts between different chains of the dimer is significantly increase when the Ag_55_ was induced. Absolutely, the dimeric Aβ_42_ peptide in binding mode with Ag_55_ forms much more of structural change than the isolated dimeric Aβ_42_. The silver nanoparticle prefers to bind to two domains, including sequences 2–5 and 35–40. The FEL of the dimer is thus altered under the influence of the nanoparticle. Among these, the number of minima has increased. The obtained results may play an important role in the searching for Aβ inhibitor pathway.

## Conflicts of interest

There are no conflicts to decare.

## Supplementary Material

RA-014-D4RA02197E-s001
