# Supplementary material for: Silver nanoparticles alter the dimerization of Aβ42 studied by REMD simulations
Source: RSC Adv. 2024 May 8;14(21):15112–9. doi: 10.1039/d4ra02197e (PMC11078207; doi:10.1039/d4ra02197e)
Supplement: RA-014-D4RA02197E-s001 [file RA-014-D4RA02197E-s001.pdf]

Electronic Supplementary Material (ESI) for RSC Advances.
This journal is © The Royal Society of Chemistry 2024

Supporting information

Silver Nanoparticle Alters the Dimerization of Aβ42 Studied by REMD simulations

Quynh Mai Thai,[ab] Phuong-Thao Tran,[c] Huong T. T. Phung,[d] Minh Quan Pham,[ef] and Son Tung Ngo,[ab*]

[a]Laboratory of Biophysics, Institute for Advanced Study in Technology, Ton Duc Thang University, Ho Chi Minh City, Vietnam

[b]Faculty of Pharmacy, Ton Duc Thang University, Ho Chi Minh City, Vietnam

[c]Hanoi University of Pharmacy, 13-15 Le Thanh Tong, Hanoi, Vietnam

[d]NTT Hi-Tech Institute, Nguyen Tat Thanh University, Ho Chi Minh City, Vietnam

[e]Institute of Natural Products Chemistry, Vietnam Academy of Science and Technology, Hanoi, Vietnam

[f]Graduate University of Science and Technology, Vietnam Academy of Science and Technology, Hanoi, Vietnam

REMD simulations were carried out with various temperatures that are arranged as 308.50, 310.12, 311.75, 313.38, 315.02, 316.66, 318.31, 319.97, 321.64, 323.30, 324.98, 326.66, 328.36, 330.06, 331.76, 333.47, 335.21, 336.93, 338.67, 340.41, 342.15, 343.91, 345.67, 347.44, 349.21, 351.00, 352.79, 354.57, 356.38, 358.19, 360.01, 361.83, 363.67, 365.51, 367.36, 369.21, 371.08, 372.95, 374.83, 376.71, 378.60, 380.50, 382.41, and 384.33.

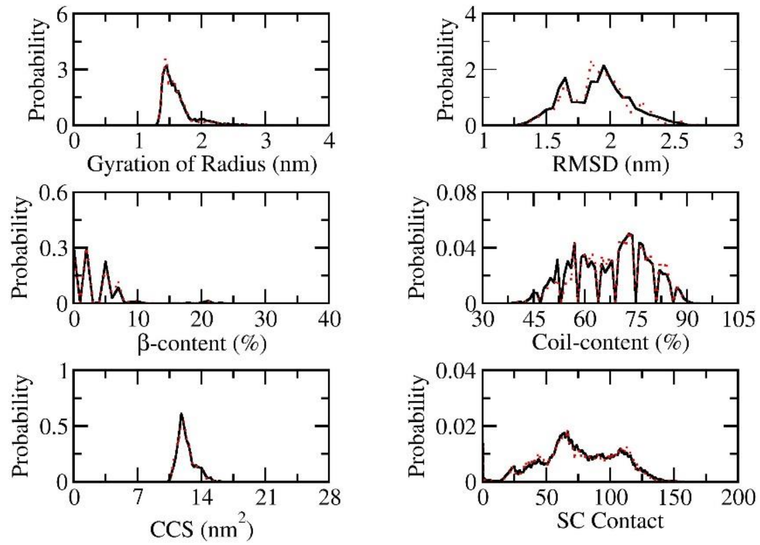

Figure S1. The superposition of computed metrics of the dimeric Aβ_{42} + Ag_{55} over the different intervals 260-500 and 380-500 ns of REMD simulations.

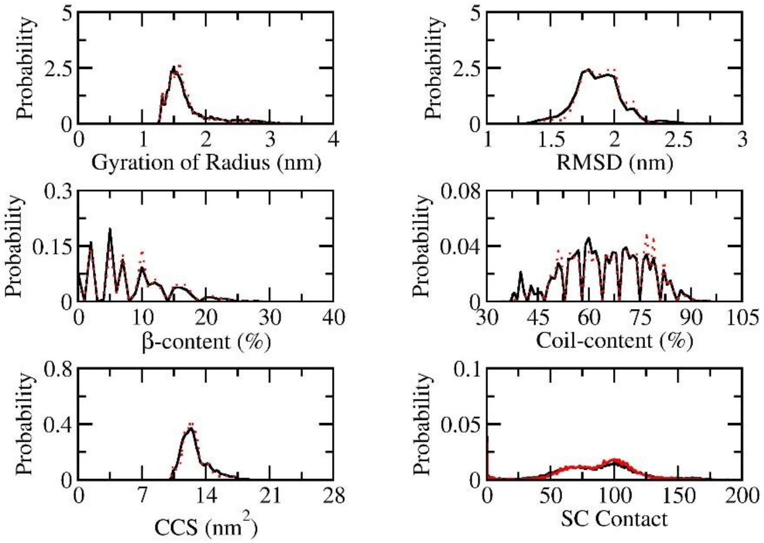

Figure S2. The superposition of computed metrics of the dimeric Aβ_{42} over the different intervals 260-500 and 380-500 ns of REMD simulations.
